# Increasing complexity: structural equivalence and combinatorial approaches to preparation of high order cocrystals

**DOI:** 10.1107/S2052252521002074

**Published:** 2021-02-24

**Authors:** Susan A. Bourne

**Affiliations:** a Centre for Supramolecular Chemistry Research, Department of Chemistry, University of Cape Town, Rondebosch 7701, Cape Town, South Africa

**Keywords:** multicomponent crystals, cocrystals, crystal engineering, structural inequivalence, combinatorial approaches

## Abstract

An algorithmic strategy to design stoichiometric quaternary and solid-solution quinary solids is described. The strategy involves recognition of structural inequivalences to generate ternary and quaternary cocrystals which can then be extended to five-component solid solutions through matching of suitable interactions.

Ever since Gerhard Schmidt (1971 ▸) proposed that a better understanding of packing principles of organic molecules would lead to predictable reactivity in their crystalline state, the topic of crystal engineering has offered the intriguing prospect of controllable functional design in the solid state. Revitalized in the 1980s by, among others, Desiraju (1989 ▸), crystal engineering has undergone explosive growth in the early years of the 21st century. This is evident in the growing number of papers on the topic in this journal (Desiraju, 2020 ▸).

From early work in the design of molecular crystal structures (MacGillivray *et al.*, 2000 ▸; Bhogala *et al.*, 2005 ▸) crystal engineering has grown and branched to include polymorphism (Broadhurst *et al.*, 2020 ▸), mechanical properties (Karki *et al.*, 2009 ▸) and improved properties of pharmaceutically or biologically active solids (Mannava *et al.*, 2021 ▸). The latter is often achieved through the formation of multi-component crystals including cocrystals. Cocrystallization has become so familiar a concept that some authors now refer to ‘cocrystal engineering’ (for example Kumar *et al.*, 2020 ▸; Ye *et al.*, 2020 ▸)

While many have explored this area, and have produced binary cocrystals for multiple applications, higher dimensional cocrystals remain relatively rare. But the challenge is an exciting one, particularly for the design of multicomponent crystals combining several active pharmaceutical ingredients (APIs). Multidrug cocrystals can be expected to produce a synergistic therapeutic effect, while crystal engineering of the molecular packing and dominant non-covalent interactions may allow the modulation of physicochemical properties to improve drug formulation and delivery. To date, the only approaches to succeed in preparing ternary API solids have been the ‘drug–bridge–drug’ strategy developed by Liu *et al.* (2018 ▸) and the ionic cocrystallization method (Mazzei *et al.*, 2019 ▸; Song *et al.*, 2020 ▸). However, the former is limited by the difficulty of identifying a suitable bridge, while ionic cocrystallization involves coordination bonds to a metal ion ‘glue’ holding the APIs together.

Increasing the number of components in a crystal vastly increases the complexity of the synthesis. An understanding of the conditions required for the reliable and reproducible formation of higher order multicomponent crystals remains elusive. This is the challenge addressed in an article in this issue of **IUCrJ** (Rajkumar & Desiraju, 2021 ▸). Earlier work by one of the authors suggested a crystal engineering strategy to achieve quaternary and quinary cocrystals, based on the structural inequivalence of some of the chemical constituents in the cocrystals (Mir *et al.*, 2016 ▸). This postulates that, if a given molecule is found in two different structural environments in a lower order cocrystal structure, these features can be exploited to increase the number of components in a new solid structure. This approach has been used to obtain ternary and quaternary cocrystals. To extend to higher order cocrystals, the authors then invoke a combinatorial approach, using the persistence of a molecule in different solid forms to select the best interactions from a range of possibilities.

The strategy is illustrated by the design and synthesis of quaternary and quinary cocrystals of model components 2-nitroresorcinol (NRES), tetramethylpyrazine (TMP), either 2,2′-bipyridine (22BP) or 2,2′-bithiophene (22TBP), and 1,2-di(4-pyridyl)ethane (DPE). A flowchart approach (see Fig. 1 ▸) is used to identify the structural inequivalences which can be exploited to extend the chain, as opposed to those that are ‘dead ends’ in the synthetic sequence. The proof of concept is shown by the synthesis of several five-component solid solution cocrystals obtained by mechanochemical combination of a quaternary cocrystal with an additional component.

Aside from the intrinsic beauty of the resulting structures, this is an elegant example of a retrosynthetic approach being applied to systems built on noncovalent interactions. While hydrogen bonding is the dominant interaction in the examples reported here, the approach lends itself to use with other forms of supramolecular interactions such as halogen bonding, coordination bonding and π–π interactions.

An algorithmic approach, such as that described here, is a positive step in the direction of developing an understanding of non-covalent interactions and their influence on synthesis of multicomponent crystals. This is essential for the advancement of crystal engineering into the new era of true design of functional materials.

## Figures and Tables

**Figure 1:**
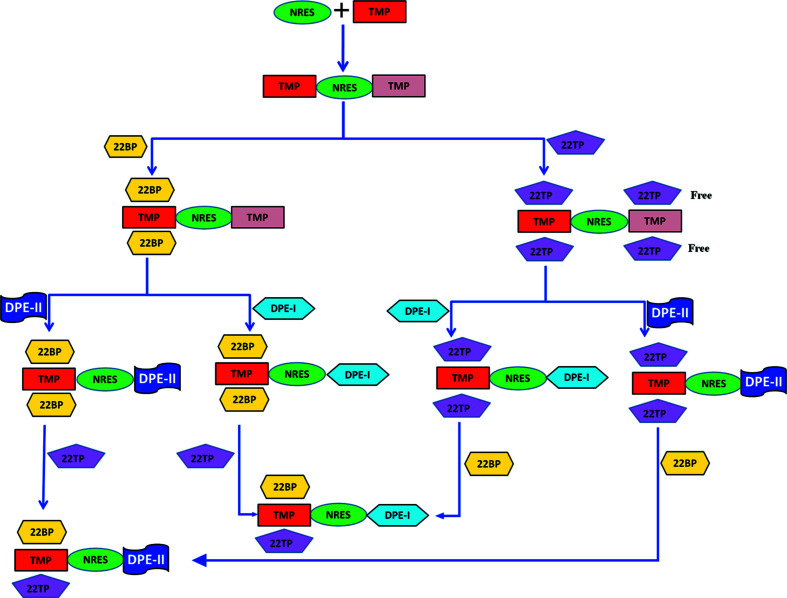
Schematic for the construction of five-component cocrystals. Reproduced from Rajkumar & Desiraju (2021 ▸)
